# Hypnotic drug use and intraoperative fluid balance associated with postoperative delirium following pancreatic surgery: A retrospective, observational, single-center study

**DOI:** 10.1371/journal.pone.0319380

**Published:** 2025-03-07

**Authors:** Zhi-Hua Huang, Jun Zhang, Xiao-ying Xu, Ying Wang, Xiao-jian Lu, Yan Luo

**Affiliations:** 1 Department of Anesthesiology, Ruijin Hospital Affiliated to Shanghai Jiao Tong University School of Medicine, Shanghai, China; 2 Department of Pancreatic Surgery, Ruijin Hospital Affiliated to Shanghai Jiao Tong University School of Medicine, Shanghai, China; Asan Medical Center, University of Ulsan College of Medicine, KOREA, REPUBLIC OF

## Abstract

**Background:**

Postoperative delirium is a common complication after various types of major surgery. The aim of this study was to identify risk factors associated with delirium following pancreatic surgery.

**Methods:**

Data from the patients who had pancreatic surgery between July 2020 and March 2021 in Ruijin Hospital affiliated with Shanghai Jiao Tong University School of Medicine were retrospectively analysed. The postoperative risk factors related to delirium were analyzed by univariate and multivariate Logistic regression analysis.

**Results:**

59 of 385 patients (15.3%) developed postoperative delirium after pancreatic surgery. The ROC curve revealed the optimal cutoff of intraoperative fluid balance was 2863ml. Furthermore, the multivariate analysis demonstrated that age ≥  65 years old [Odds ratio (OR) 2.01; 95% Confidence interval (CI) 1.12-3.63; p =  0.019], hypnotic drug use (OR 4.17; 95% CI 1.50-11.10; *p* =  0.005), and intraoperative fluid balance (OR 2.57; 95% CI 1.37-4.84; *p* =  0.003) were the independent risk factors of postoperative delirium.

**Conclusion:**

This study identified that intraoperative fluid balance and hypnotic drug use were independent risk factors associated with postoperative delirium development after pancreatic surgery.

## Introduction

Patients with pancreatic malignant and benign diseases often receive major surgery, namely pancreaticoduodenectomy, distal pancreatectomy or total pancreatectomy. Postoperative complications such as bleeding, pancreatic fistula, infection, gastroparesis and among others are often following this type of major surgery [1–5].

Postoperative delirium often occurs after major surgery [6,7] and leads to a high rate of other postoperative complications, longer hospital stays, and increased hospital costs [8,9]. A number of previous studies reported that various risk factors related to postoperative delirium occurrence include advanced age, history of hypnotic drugs, preoperative cognitive decline, preoperative increased alcohol intake, lower preoperative hemoglobin levels, history of neurologic comorbidity, depression, drug use, and cumulative fluid balance [10–15] although risk factors for postoperative delirium vary with the type of surgery. However, study into risk factors associated with postoperative delirium after pancreatic surgery is very limited. Only several previous researches with small size identified older age and history of hypnotic drugs (specifically benzodiazepines) to be potential risk factors [16–19].

This study was designed to identify postoperative delirium risk factors in patients who underwent pancreatic surgery. The ultimate aim of this study is to improve perioperative management for better recovery in this high-risk surgery of patients.

## Materials and methods

### Ethical statement

This retrospective, single-center study was approved by the Ethics Committee of Ruijin Hospital affiliated with Shanghai Jiao Tong University School of Medicine and was registered in the Chinese Clinical Trial registry (ChiCTR2100047405). Given the retrospective nature, informed consent forms were waived.

### Patients

This study included patients who were over 18 years old and underwent radical pancreatic surgery at Shanghai Ruijin Hospital between July 2020 and March 2021. The inclusion criteria were: 1) no communication barrier and good cooperation; 2) no history of serious cardiovascular and cerebrovascular diseases; 3) no history of delirium; 4) undergoing major pancreatic surgery and 5) no occurrence of serious cardiovascular adverse events during the operation. The exclusion criteria were: 1) surgery type being exploratory operation and local excision; 2) immediate transfer to Intensive Care Unit (ICU) for further treatment after the operation; 3) reoperation; 4) declining postoperative surveillance; and 5) incomplete medical data record.

### Laboratory examinations and data collection

Patients’ demographics including sex, age, alcohol and smoking history, depression, history of stroke, sleep disorders, history of hypnotic drugs, education time together with laboratory data including albumin, glucose, total bilirubin, blood urea nitrogen (BUN) and creatinine were collected. Furthermore, hypnotic drug use was defined as the use of medications within a certain period before surgery, this included long-term use, short-term use and irregular use, aimed at helping patients alleviate preoperative anxiety and treat insominia.

Biliary drainage, disease pathology, and intraoperative parameters such as surgical approach, surgery type, operation time, estimated blood loss, and intraoperative fluid balance were obtained from their medical records. Specifically, the intraoperative fluid balance refers to the net change in a patient’s body fluids during surgery, calculated by determining the difference between the total volume of fluids administered and the total volume of fluids lost. Other postoperative complication including postoperative pancreatic fistula (POPF), biliary fistula, intestinal fistula, delayed gastric emptying (DGE), intra-abdominal infection, post-pancreatectomy hemorrhage (PPH) and 30-day mortality were collected.

### Diagnosis of postoperative delirium

In our hospital, all patients were assessed with the Confusion Assessment Method (CAM)[20] for postoperative delirium (POD) twice per day up to postoperative day 7. The POD incidence was also harvested. Accordingly, patients were then divided into two groups based on the presence or absence of delirium for data analysis and comparison.

All data used in this research were collected prior to statistical analysis, with the collection period spanning from July 2020 to March 2021.

### Statistical analysis

Chi-square test or Fisher precision test were used to analyse categorical variables. The continuous variables are expressed as mean ± standard deviation (SD) if normally distributed and analysed with Student’s t-test or otherwise, data were expressed as median and interquartile range (IQR) and was analyzed with Mann-Whitney U test. Receiver-operator characteristics (ROC) curve analysis was used to determine the optimal cutoff value of intraoperative fluid balance. Finally, all variables were significant in univariate analyses (p <  0.05) were entered into the binary logistic regression. Odds ratios (ORs) and 95% confidence intervals (CIs) were also obtained. The statistical power of our study was carefully considered during the design phase to ensure that we could reliably detect clinically significant effects, particularly in relation to delirium. To estimate the power of our study, we conducted a power analysis prior to data collection. Based on the expected effect sizes from previous similar studies and the target significance level (α =  0.05), we calculated the required sample size to achieve an acceptable power (over 90%) for detecting key outcomes, such as the association between intraoperative fluid balance and postoperative delirium. R version 4.1.3. was used for all statistical analysis. A P value less than 0.05 (two-tailed) was considered to be statistically significant.

## Results

### Demographics and clinical characteristics

**Fig 1:**
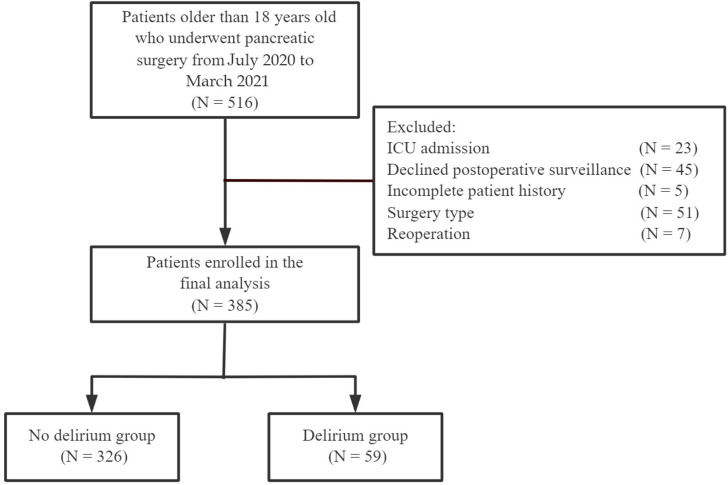
Flow chart of patients.

Between July 2020 and March 2021, a total of 516 patients aged 18 years and above who underwent radical pancreatic surgery were screened and the data from 385 cases were included in the study (Fig 1). Out of the 385 patients, 59 (15.3%) developed postoperative delirium. The baseline clinical characteristics of the patients are presented in Table 1. There were 202 males (52.5%) and 183 females (47.5%). The majority of patients had no alcohol or smoking history. The postoperative delirium patients were older (p =  0.020) and had a higher proportion of hypnotic drug use (p =  0.002), however, there were no significant differences in the baseline characteristics between the two groups.

**Table 1:** Baseline clinical characteristics of included patients and stratified by the status of postoperative delirium.

	Overall (n = 385)	No delirium (n = 326)	Delirium (n = 59)	P
Sex				0.58
Male	202 (52.5)	173 (53.1)	29 (49.2)	
Female	183 (47.5)	153 (46.9)	30 (50.8)	
Age (years)	58.33 (13.41)	57.66 (13.64)	62.05 (11.44)	0.02
Alcohol history	47 (12.2)	43 (13.2)	4 (6.8)	0.166
Smoking history	64 (16.6)	57 (17.5)	7 (11.9)	0.286
Depression	22 (5.7)	16 (4.9)	6 (10.2)	0.109
History of stroke	42 (10.9)	32 (9.8)	10 (16.9)	0.106
Sleep disorders	112 (29.1)	91 (27.9)	21 (35.6)	0.232
Hypnotic drug use	20 (5.2)	12 (3.7)	8 (13.6)	0.002
Education time (years)				0.572
<6	83 (21.6)	70 (21.5)	13 (22.0)	
6-12	196 (50.9)	163 (50.0)	33 (55.9)	
> 12	106 (27.5)	93 (28.5)	13 (22.0)	

SD: standard deviation; Education time: the years of formal education, < 6 years: Primary education or less (e.g., elementary school); 6-12 years: Secondary education (e.g., middle and high school); > 12 years: Higher education (e.g., college, university, and beyond).

Preoperative laboratory data did not have any significant impacts on postoperative delirium. A total of 242 (58.2%) patients were diagnosed with malignant tumors, while 161 (41.8%) were diagnosed with benign tumors (Table 2).

**Table 2:** Preoperative parameters and pathological data of patients stratified by the status of postoperative delirium.

Variables	No delirium (n = 326)	Delirium (n = 59)	P
Anemia	93 (28.5)	21 (35.6)	0.274
Albumin (g/L)			0.372
≥35	299 (91.7)	52 (88.1)	
<35	27 (8.3)	7 (11.9)	
Glucose (mmol/L)	5.53 (4.86 to 6.50)	5.64 (5.03 to 6.65)	0.26
Total bilirubin (μmol/L)	13.50 (9.83 to 19.35)	14.40 (11.80 to 21.20)	0.127
BUN (mmol/L)	5.00 (4.10 to 6.00)	5.00 (4.00 to 5.75)	0.683
Creatine (μmol/L)	69.00 (60.00 to 82.00)	72.00 (60.00 to 83.50)	0.458
PBD	31 (9.5)	4 (6.8)	0.659
Pathology			0.104
Malignant	184 (56.4)	40 (67.8)	
Benign	142 (43.6)	19 (32.2)	

BUN: blood urea nitrogen; PBD: preoperative biliary drainage; IQR: interquartile range. Anemia: hemoglobin levels below the criteria for Chinese adults, adult males < 120 g/L, adult females (non-pregnant) < 110 g/L.

### Perioperative parameters

Table 3 displays intraoperative parameters and anesthesia data collected from the 385 patients who underwent radical pancreatic surgery. Of these patients, 268 (69.6%) underwent open surgery (OS) and 117 (30.4%) underwent minimally invasive surgery (MIS). The most common surgery type was pancreaticoduodenectomy (PD) (53.2%), followed by distal pancreatectomy (DP) (38.2%), total pancreatectomy (TP) (4.7%), and middle pancreatectomy (MP) (3.9%). The operation time was longer than 180 minutes in 74% of patients with no significant difference observed between the delirium and no delirium groups (p =  0.086). The surgery type, estimated blood loss (EBL) and other anesthesia outcomes were not significantly different between the two groups (Table 3).

**Table 3:** Intraoperative parameters and anesthesia data of patients stratified by the status of postoperative delirium.

Variables	No delirium (n = 326)	Delirium (n = 59)	P
Surgical approach			0.227
OS	223 (68.4)	45 (76.3)	
MIS	103 (31.6)	14 (23.7)	
Surgery type			0.883
PD	171 (52.5)	34 (57.6)	
DP	127 (39.0)	20 (33.9)	
TP	15 (4.6)	3 (5.1)	
MP	13 (4.0)	2 (3.4)	
Operation time (minutes)			0.086
<180	90 (27.6)	10 (16.9)	
≥180	236 (72.4)	49 (83.1)	
EBL (ml)	300.00 (200.00 to 600.00)	300.00 (200.00 to 600.00)	0.507
Tansfusion	203 (62.3)	39 (66.1)	0.575
Anesthetic gas			0.786
DES	312 (94.8)	56 (93.2)	
SEV	14 (4.3)	3 (5.1)	
Intraoperative MIZ	247 (75.8)	39 (66.1)	0.118
Sufentanyle (ug/kg)	1.00 (0.82 to 1.17)	1.06 (0.90 to 1.23)	0.05
Remifentanil (mg/kg)	1.00 (0.50 to 2.00)	1.20 (0.50 to 2.00)	0.551
Intraoperative infusion (ml)	3350.00 (2662.50 to 4400.00)	4100.00 (2900.00 to 4825.00)	0.022
Urine output (ml)	600.00 (312.50 to 1000.00)	700.00 (400.00 to 1050.00)	0.435
Intraoperative fluid balance (ml)	2350.00 (1850.00 to 2850.00)	2700.00 (2100.00 to 3250.00)	0.007

OS: open Surgery; MIS: minimally invasive surgery; PD: pancreaticoduodenectomy; DP: distal pancreatectomy, TP: total pancreatectomy; MP: middle pancreatectomy; EBL: estimated blood loss; DES: desflurane; SEV: sevoflurane; MIZ: midazolam; IQR: interquartile range.

**Fig 2:**
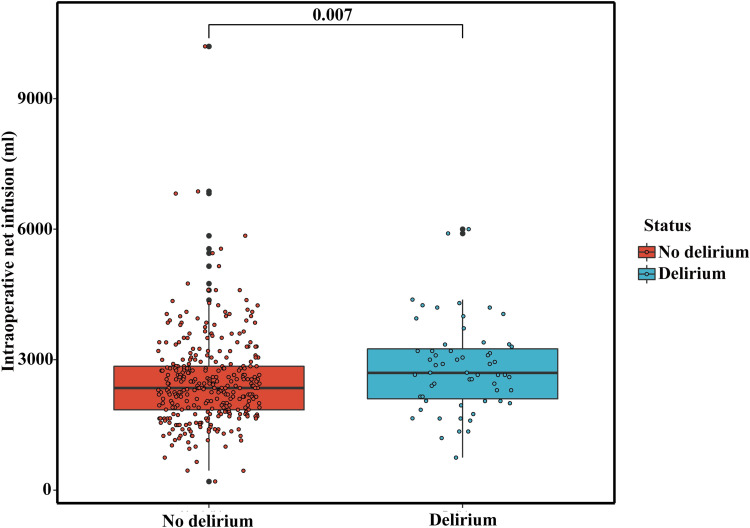
Comparation of intraoperative fluid balance between the delirium group and the no delirium group.

Patients who developed postoperative delirium had higher intraoperative dosages of sufentanyle (p =  0.050), intraoperative fluid infusion (p =  0.022) and fluid balance (p =  0.007) compared to those who did not develop delirium (Fig 2). The postoperative complications including postoperative pancreatic fistula (POPF), biliary fistula, intraabdominal infection, post-pancreatectomy hemorrhage (PPH) and 30-day mortality did not show any significant differences between the delirium group and no delirium group (S1 Table).

### Risk factors of postoperative delirium

The ROC of intraoperative fluid balance and POD was shown in S1 Fig. The area under the curve (AUC) was 0.609. Although this model has a low sensitivity, it boasts a high specificity, which makes it generally quite satisfactory. The optimal cut-off value for the intraoperative fluid balance estimated with the ROC curve is 2863 ml. In addition, univariate analysis showed that the older age, hypnotic drug use, the higher intraoperative dosage of sufentanyle, the longer operation time, and the higher intraoperative fluid balance were the risk of postoperative delirium. Further multivariate logistic regression analysis revealed that age ≥  65 years old (OR 2.01; 95% CI 1.12-3.63; p =  0.019), a history of hypnotic drug use (OR 4.17; 95% CI 1.50-11.10; p =  0.005), and intraoperative fluid balance ≥ 2863 ml (OR 2.57; 95% CI 1.37-4.84; p =  0.003) were the independent risk factors for POD after pancreatic surgery (Table 4).

**Table 4:** Univariate analysis and multivariate analysis of risk factors for postoperative delirium.

Risk factor	Univariate analysis			Multivariate analysis		
	OR	CI	P	OR	CI	P
Age, ≥ 65 years vs. < 65 years	2.26	1.29-3.97	<0.001	2.01	1.12-3.63	0.019
Hypnotic drug use, yes vs. no	4.1	1.60-10.53	0.002	4.17	1.50-11.10	0.005
Sufentanyle, ≥ 1 ug/kg vs. < 1 ug/kg	1.83	1.03-3.28	0.04	1.39	0.71-2.82	0.347
Intraoperative fluid balance, high vs. standard ml	2.55	1.44-4.51	<0.001	2.57	1.37-4.85	0.003
Operation time, ≥ 180 minutes vs. < 180 minutes	1.87	0.91-3.85	0.086	1.07	0.46-2.64	0.874

Intraoperative fluid balance, based on the Youden index’s cutoff value derived from the ROC curve analysis, high if exceeding the Youden index cutoff value from the ROC analysis, standard otherwise.

## Discussion

Our study found that 15.3% of patients developed delirium after pancreatic surgery, which is slightly lower than previous studies reported [16,18]. We identified older age, a history of hypnotic drug use, and higher intraoperative fluid load as independent risk factors for postoperative delirium.

It is important to note that major abdominal surgeries, including pancreatic surgery, are typically performed on elderly patients [21–23]. However, our study included a broader age range (18 years to elderly), which reflects the real-world clinical population. Despite this, our findings are consistent with previous study indicating that old age and a history of hypnotic drug use are risk factors for postoperative delirium. Furthermore, our study highlights the importance of managing intraoperative fluid balance to reduce the risk of delirium after pancreatic surgery.

Risk factors associated with postoperative delirium in surgical patients had been reported previously. For instance, an analysis by Tomimaru et al. [16] indicated that age as well as a history of benzodiazepine use were predictive factors of postoperative delirium among patients underwent pancreaticoduodenectomy. Similarly, Chaiwat et al. [24] investigated the impact of age and perioperative use of benzodiazepine on postoperative delirium in critically ill surgical patients and observed that there was a correlation between older age, use of benzodiazepine and postoperative delirium. Hiraki et al. [25] also reported that older age (over 80 years old) and preoperative use of sleeping pills were risk factors for early postoperative delirium following laparoscopic colorectal surgery in the elderly. Consistent with these findings, our study found that patients who developed postoperative delirium after pancreatic surgery were older and had a higher proportion of hypnotic drug use history. Indeed, our multivariate analysis showed that the odds of postoperative delirium were twice as high for patients aged ≥ 65 years compared to younger patients. Additionally, the odds were four times higher for patients with a history of hypnotic drug use.

Furthermore, we noted that patients with a high positive intraoperative fluid load had twice high more likely to develop postoperative delirium after pancreatic surgery. These findings are consistent with previous surveys. Mailhot et al. [12] reported that patients with postoperative delirium after cardiac surgery had a higher cumulative fluid balance. Additionally, Zhang et al. [26] found that in elderly patients undergoing spinal stenosis surgery, the higher fluid intake, the more likely to develop postoperative delirium. Therefore, our investigation further confirms the importance of fluid management in reducing the possibility of postoperative delirium, particularly in high-risk surgical patients [27].This may be due to several mechanisms, such as capillary leakage and interstitial edema, inflammatory response in the nervous system, hypoxemia, and electrolyte imbalance [28–30].

Age and a history of hypnotic drug use are invariable indicators of postoperative delirium. Patients with these risk factors should receive preoperative consultation to anticipate the services required after delirium may occur. Standardized perioperative programs can significantly reduce the incidence and severity of delirium. Intraoperative fluid balance can be promptly assessed and managed to decrease the postoperative delirium risk.

While EBL did not reach statistical significance in our multivariate models for postoperative delirium, proper hemostasis remains a crucial factor in minimizing complications during and after major surgeries, including pancreatic operations. Minimizing blood loss can reduce the need for transfusions, which have been shown in several studies to increase the risk of adverse outcomes, including infections, prolonged recovery, and cognitive dysfunction such as delirium.

There are several limitations in this study. Firstly, it is a retrospective study conducted at only one institution. Secondly, the duration of the study was limited to 7 days, which may have resulted in an underestimation of the incidence of delirium. Thirdly, delirium subtypes were not captured in this study, it is an important area for future research.

In conclusion, our findings highlight the importance of managing intraoperative fluid load to decrease the risk of postoperative delirium in patients following pancreatic surgery. Additionally, preoperative consultation and standardized perioperative programs should be implemented to reduce the occurrence and severity of postoperative delirium, particularly in patients with risk factors such as advanced age and a history of hypnotic drug use. Further research will be needed to explore the underlying mechanisms of postoperative delirium and identify effective preventive strategies.

## Supporting information

S1 Fig The ROC curve for postoperative delirium after pancreatic surgery. (The AUC was 0.609.)
(TIF)

S1 Table Postoperative complications and 30-day mortality. (DOCX)

S2 Table List of abbreviations. (DOCX)
