# Supplementary figures and images for: Hypnotic drug use and intraoperative fluid balance associated with postoperative delirium following pancreatic surgery: A retrospective, observational, single-center study
Source: PLoS One. 2025 Mar 7;20(3):e0319380. doi: 10.1371/journal.pone.0319380 (PMC11888130; doi:10.1371/journal.pone.0319380)

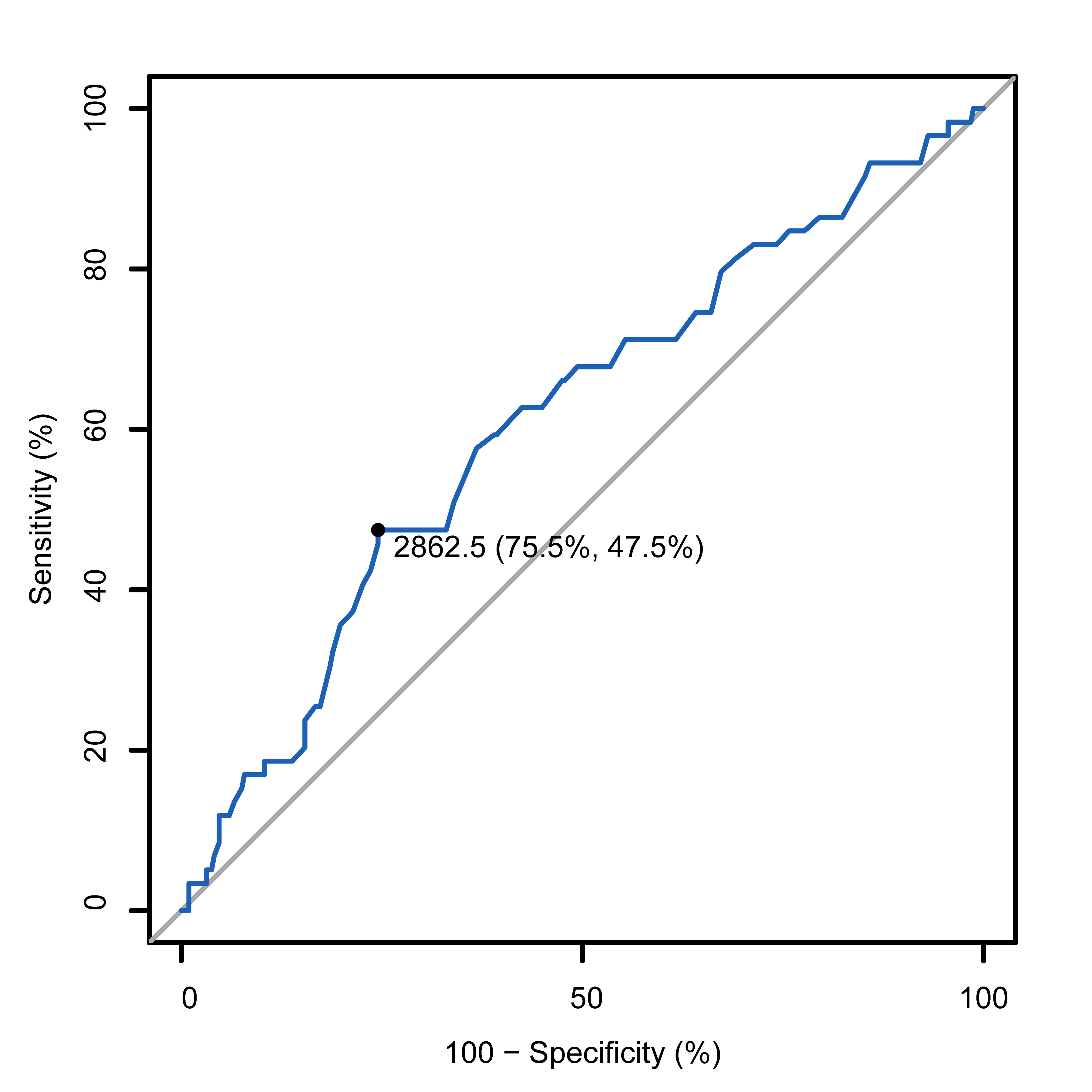

Supplement: S1 Fig — (The AUC was 0.609.) (TIF) [file pone.0319380.s001.tif]
