# Supplementary material for: Hypnotic drug use and intraoperative fluid balance associated with postoperative delirium following pancreatic surgery: A retrospective, observational, single-center study
Source: PLoS One. 2025 Mar 7;20(3):e0319380. doi: 10.1371/journal.pone.0319380 (PMC11888130; doi:10.1371/journal.pone.0319380)
Supplement: S1 Table — (DOCX) [file pone.0319380.s002.docx]

**Supporting Information**

**S1 Table. Postoperative complications and 30-day mortality**

|  | **Overall (n = 385)** | **No delirium(n = 326)** | **Delirium (n = 59)** | **P** |
| --- | --- | --- | --- | --- |
| **POPF** | 72 (18.7) | 63 (19.3) | 9 (15.3) | 0.461 |
| **Biliary fistula** | 12 (3.1) | 12 (3.7) | 0 (0.0) | 0.132 |
| **Intestinal fistula** | 1 (0.3) | 1 (0.3) | 0 (0.0) | 0.670 |
| **DGE** | 2 (0.5) | 1 (0.3) | 1 (1.7) | 0.172 |
| **Intraabdominal infection** | 113 (29.4) | 91 (27.9) | 22 (37.3) | 0.146 |
| **PPH** | 6 (1.6) | 6 (1.8) | 0 (0.0) | 0.294 |
| **Reoperation** | 22 (5.7) | 18 (5.5) | 4 (6.8) | 0.702 |
| **30-day mortality** | 15 (3.9) | 13 (4.0) | 2 (3.4) | 0.827 |

DGE, delayed gastric emptying; POPF, postoperative pancreatic fistula; PPH, post-pancreatectomy hemorrhage.
