# Supplementary material for: Hypnotic drug use and intraoperative fluid balance associated with postoperative delirium following pancreatic surgery: A retrospective, observational, single-center study
Source: PLoS One. 2025 Mar 7;20(3):e0319380. doi: 10.1371/journal.pone.0319380 (PMC11888130; doi:10.1371/journal.pone.0319380)
Supplement: S2 Table — (DOCX) [file pone.0319380.s003.docx]

**Supporting Information**

**S2 Table. List of abbreviations**

| **Abbreviation** | **Full name** |
| --- | --- |
| **ROC** | Receiver-operator characteristics |
| **OR** | Odds Ratio |
| **CI** | Confidence Interval |
| **PD** | Pancreaticoduodenectomy |
| **DP** | Distal Pancreatectomy |
| **TP** | Total Pancreatectomy |
| **BUN** | Blood Urea Nitrogen |
| **IQR** | Interquartile Range |
